# An Investigation of Nile Tilapia (*Oreochromis niloticus*) Movement Trajectories Under Ammonia Stress Using Image Processing Techniques

**DOI:** 10.3390/life15071004

**Published:** 2025-06-24

**Authors:** Muhammed Nurullah Arslan, Güray Tonguç, Beytullah Ahmet Balci, Tuba Sari

**Affiliations:** 1 Department of Aquaculture, Faculty of Fisheries, Recep Tayyip Erdoğan University, Rize 53100, Türkiye; nurullah.arslan@erdogan.edu.tr; 2 Department of Management Information Systems, Faculty of Applied Sciences, Akdeniz University, Antalya 07070, Türkiye; guraytonguc@akdeniz.edu.tr (G.T.); tubasari@akdeniz.edu.tr (T.S.); 3 Department of Aquaculture, Faculty of Fisheries, Akdeniz University, Antalya 07070, Türkiye

**Keywords:** aquaculture, ammonia, fish behaviors, image processing, Nile Tilapia, non-invasive, object tracking

## Abstract

This study examined the behavioral responses of Nile Tilapia (*Oreochromis niloticus*), a key aquaculture species, to ammonia stress using non-invasive image processing techniques. The experiment was conducted under controlled laboratory conditions and involved four groups exposed to ammonium chloride concentrations (0, 100, 200, and 400 mg·lt^−1^). Movement trajectories of individual fish were recorded over 10 h using high-resolution cameras positioned above and beside glass tanks. Images were processed with the Optical Flow Farneback algorithm in Python, implemented in Visual Studio Code with OpenCV and NumPy libraries, achieving a 91.40% accuracy rate in tracking fish positions. The results revealed that increasing ammonia levels restricted movement areas while elevating movement irregularity and activity. The 0 mg·lt^−1^ group utilized the glass tank homogeneously, covering 477 m. In contrast, the 100 mg·lt^−1^ group showed clustering in specific areas (796 m). At 200 mg·lt^−1^, clustering intensified, particularly along the glass tank’s left edge (744 m), and at 400 mg·lt^−1^, fish exhibited severe restriction near the water surface with markedly increased activity (928 m). Statistical analyses using Kruskal–Wallis and Dunn tests confirmed significant differences between the 400 mg·lt^−1^ group and others. No difference was observed between the 0 mg·lt^−1^ and 100 mg·lt^−1^ group, indicating tolerance to lower concentrations. The study highlights the importance of ammonia levels in water quality management and reveals the potential of image processing techniques for automation and stress monitoring in aquaculture.

## 1. Introduction

The global population increases the need for alternative food sources every day. The aquaculture sector is indispensable in terms of food security and the economy. It is one of the fastest-growing sectors in the world, but it faces challenges such as the decrease in fish resources and the tightening of international standards. Despite this, aquaculture has managed to steadily increase fish production, but high technical requirements limit growth. Smart fishing applications make the sector more efficient with innovations such as automatic sensors, feeding systems, and real-time monitoring. Yet, technologies for monitoring fish behavior in real time are still needed [1,2,3,4].

During intensive aquaculture, commercial feed that is not consumed by fish changes the water quality by turning into suspended compounds such as ammonia, nitrate, phosphate, carbon dioxide, and organic matter [5]. Toxic nitrogen levels containing ammonia often cause fish deaths, while in the long term, stress weakens the immune system, increases susceptibility to diseases, reduces growth rate, reduces reproductive capacity, and increases mortality [6,7,8]. All these stress-induced changes are associated with differences in fish behavior [9,10,11,12,13,14,15].

Early detection of stress parameters allows farmers to take rapid and mitigating measures to minimize production losses [16]. Environmental changes can rapidly increase hormonal stress parameters. Therefore, behavioral monitoring methods such as fluorescent markers or video-based observation have been developed to determine the location of fish and study their behavior [17,18]. In recent years, computer technologies have been used to detect stress in fish with continuous, in situ, non-invasive, and non-contact measurement methods [4,15,16,19,20].

While previous studies focused on physiological responses in species like goldfish [20], behavioral analyses have been limited. Additionally, research has explored individual/swarm movements [20]. Research has also demonstrated image processing as a non-invasive tool to monitor behavior and stress in aquaculture [4].

Computer vision techniques provide automatic, non-invasive, and cost-effective methods to assess behavioral changes, enabling the determination of stress levels in aquatic animals [17,21,22,23,24,25]. Video-based analysis records fish movements and enables quantitative analysis of these images. This allows studying fish behavior more efficiently and accurately [26,27,28,29,30]. In addition, this analysis can quickly detect any abnormalities and contribute to improving aquaculture safety. Analysis of real-time fish school video streaming data can improve aquaculture efficiency, reduce labor costs, and further modernize fishery production by automating and optimizing smart feeding processes [31].

Nile Tilapia (*Oreochromis niloticus*) is the second most commercially farmed fish species in the world after carp (*Cyprinus carpio*), with production reaching 6.9 million tons in 2020 [25,32,33,34]. This increase is due to the species’ superior traits, such as rapid growth, high reproductive capacity, resistance to dense stocking, and strong marketability [33,35]. Studies on Nile Tilapia provide valuable information in understanding their behavior by revealing physiological and biochemical stress responses to ammonia exposure. Ref. [36] revealed that acute ammonia exposure increased heart rate variability and reduced metabolic scope. Ref. [37] detected that dietary interventions such as increasing protein intake reduced some of the deleterious effects of chronic ammonia exposure, highlighting dietary modifications as a potential strategy to support fish health under stress conditions. The interaction of environmental factors such as ammonia and salinity has been shown to significantly alter antioxidative responses, biochemistry, and immunity [38].

In this study, the effects of different ammonia concentrations on tilapia fish were investigated using non-invasive image analysis techniques, and individual behavioral responses to ammonia stress were revealed.

## 2. Materials and Methods

**Experimental Setup**

**Figure 1:**
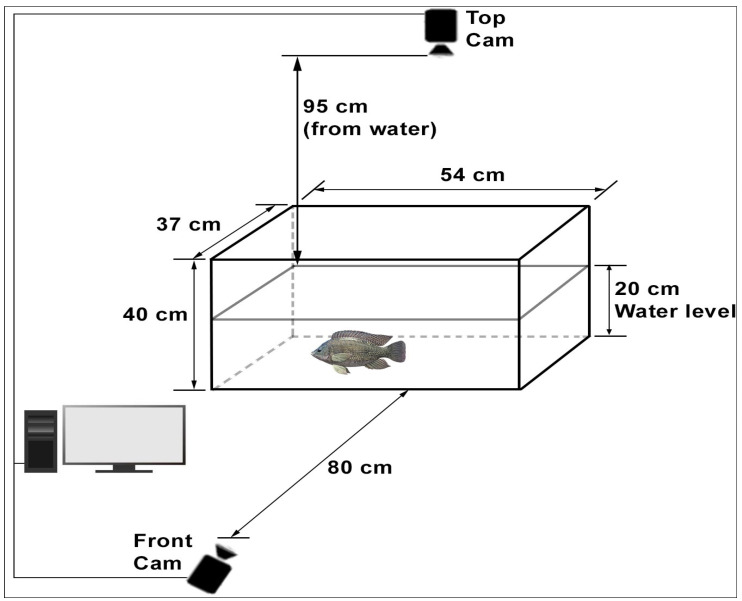
The experimental setup for monitoring Nile Tilapia behavior in the glass tanks.

In this study, the process of examining the fish by taking photographs was applied as a method that does not harm the fish (non-invasive) and does not affect its natural state in its habitat. The experimental setup consisted of glass tanks, each measuring 54 × 37 × 40 cm (Figure 1). Two high-resolution cameras providing front and top views were mounted in each glass tank [4,20]. The front camera was placed 80 cm from the glass tank, and the top camera was placed 95 cm from the water level. When determining these distances, care was taken to ensure that the camera could fully see the glass tank surface. Considering the size of the fish, the height at which the fish can move freely horizontally and vertically was defined as 20 cm. The system was supported by appropriate lighting, a central air pump, and the necessary software components.

**Fish and Experimental Design**

In the study, six Nile Tilapia (*Oreochromis niloticus*) of similar length and weight were used (Table 1). Experiments were carried out with three different ammonia concentrations (100, 200, and 400 mg·lt^−1^) under controlled laboratory conditions, and each experiment lasted two days with two replicates [4,20]. The control medium was maintained on the first day, and ammonium chloride (NH_4_Cl) was added on the second day. Ammonium concentration was stable during the experiment, as no water change was made. Water quality parameters (temperature, pH, oxygen, ammonia, ammonium, nitrite, and nitrate) were measured in the morning (08:55) and evening (19:05) using the Hach-Lange HQ40d instrument (Ames, IA, USA) and Sera aqua-test kits (Heinsberg, Germany) (Table 2).

**Image Acquisition and Processing**

During the shooting, glass tanks were photographed every second using cameras and third-party software (Bandicam, 6.0.4.2024). In this way, image acquisition is aimed at monitoring the movements of the fish almost without loss. Approximately 865 thousand images were obtained by performing two repetitions for six days and ten hours (between 09:00–19:00). The images were obtained with 1920 × 1080 resolution. The date and time information of each captured image was determined as the name of that image. The obtained images were processed in line with the algorithm developed for this study in the Visual Studio Code (1.98.2) software development environment using OpenCV (OpenCV-Python 4.8.0.74), NumPy (1.23.0) and matplotlib (3.7.1) software libraries, and Python (3.9.13) codes. In the study, an attempt was made to track the fish in the software using a single fish in the images [4,20]. In the images taken from the front camera, the long side was evaluated as x and the short side as y; in the images taken from the upper camera, the long side was evaluated as x and the short side as z. The x-coordinate information of the fish detected in the images taken simultaneously by both the front and upper cameras was matched and evaluated. As a result of processing each image, the date–time of the image, the x and y coordinates of the fish (in pixels), the area covered by the fish in the image, and the angle of the fish were obtained. In addition, using these data, the time elapsed since the beginning of the experiment, the x, y, and z coordinates of the fish in mm, and the swimming distance of the fish between the two images were produced. When calculating the distance between two points in a three-dimensional environment, the formula given below, which is the three-dimensional adaptation of the Euclidean distance formula, was used.

$$d=\sqrt{{\left(x2-x1\right)}^{2}+{\left(y2-y1\right)}^{2}+{\left(z2-z1\right)}^{2}}$$

**Figure 2:**
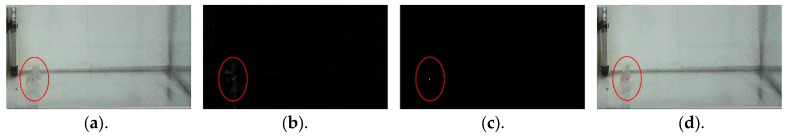
Processing steps of images taken from the camera. ((**a**). Cropped image toe processed, (**b**). Differenced image, (**c**). Thresholded image, (**d**). Contour is detected and center point is marked image).

In the given formula, x1, y1, and z1 indicate the initial position of the fish; x2, y2, and z2 indicate the subsequent position of the fish; and d indicates the distance between two points. In the developed software, all images to be processed are given to the software via the file selection window, and these files are kept in a list in the software. In the algorithm in the software, each image is first opened one by one in a loop. Each opened image is cropped according to the habitat dimensions of the fish determined by the researchers to shorten the processing time and prevent erroneous information from being obtained and converted to grayscale. From each image, the cropped and grayscale image obtains 2 frames after it is extracted. The difference image is thresholded with a value of 60 and converted to a black-and-white image. This value was obtained because of various experiments. Contours on the thresholded difference image are detected with the findContours command in the OpenCV library and marked on the image. The contour with the largest area is determined from the contours observed; thus, erroneous detections caused by fluctuations or air bubbles in the water are eliminated, and only the area covered by the fish in the image is determined. The center points of the obtained area are detected with the OpenCV moments command, and the information obtained at the end of each cycle is logged into the csv file (Figure 2).

The random sampling method was used for the accuracy analysis of the image processing algorithm developed by the researchers [39,40,41]. Our data set includes approximately 36.000 images from each experimental group. Since the data density is similar in all time zones, the simple random sampling method was preferred in order not to create a systematic bias. A sampling process was carried out with the NumPy library, where each image was selected with equal probability, the necessary changes were made in the software where the images were analyzed, random files were selected from the data set, and the researchers manually verified the accuracy of the fish detection made on each selected image.

**Statistical Analysis**

The fish position and movement data obtained in the study were also examined statistically. Since the fish movement data taken every second would not be suitable for this process due to their frequency, 10-second speed averages were taken, and 3600-speed information was analyzed for each experiment. For the normality test of the measured values, the one-sample Kolmogorov–Smirnov test [42] was used, and the Kruskal–Wallis test, which is a non-parametric test, was used for differences between groups [43]. Since a significant difference was detected between the groups using the Kruskal–Wallis test, a post hoc test was applied to show which groups were different [4,44]. Statistical analyses were performed with Python codes on the Google Colab platform at a confidence interval of 95% (*p* < 0.05) [45].

## 3. Results

In the study using Nile Tilapia (*O. niloticus*), a setup consisting of four glass tanks was established. One fish was placed in each glass tank, and one glass tank was determined as the 0 mg·lt^−1^ group, while the other three glass tanks were added with ammonium chloride at concentrations of 100, 200, and 400 mg·lt^−1^, respectively. A camera was placed in each glass tank to see from the side and top, and images were taken from all cameras once a second for 10 h (between 09:00 and 19:00). As a result of processing the images, the location information of the fish was obtained based on time. The 0 mg·lt^−1^ group was compared with the other experimental groups, and the potential effects of the presence of ammonium chloride in the water environment on the organisms of the fish were evaluated. The water quality measurements presented in Table 2 compare the values before (day 1) and after (day 2) the addition of ammonium chloride.

**Movement Trajectories**

**Figure 3:**
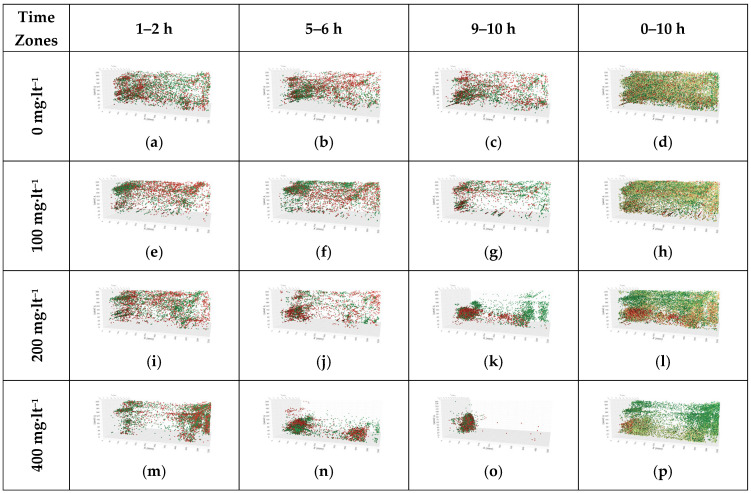
3D coordinate distributions of experimental groups (perspective views from the front top corner of the glass tanks).(Green dots represent the points where the fish were in the early hours of the relevant time zone, and red dots represent the later hours).

In Figure 3, three-dimensional representations of the location information obtained from the analysis of fish images are given by organizing them according to the experimental groups and time intervals. In the three-dimensional scatter plots, green dots represent the points where the fish were in the early hours of the relevant time zone, and red dots represent the later hours (Figure 3). The data of the 0 mg·lt^−1^ group are shown in Figure 3a–d without the addition of ammonium chloride. Here, when the 10-hour observation and the examinations conducted at 2-h intervals are evaluated, it is seen that the fish are in every area of the glass tank (Figure 3a–c). The fish movements were detected to be spread over a wider area of the glass tank. This situation can be interpreted as the fish exhibiting normal behaviors in the absence of a stress factor. In Figure 3d, the coordinates where the fish were located for 10 h are presented. The data of the glass tank to which 100 mg·lt^−1^ ammonium chloride was added are given in Figure 3. Accordingly, while the fish were moving around homogeneously in every part of the glass tank in the first hours (1–2 h, Figure 3e), it was observed that the movements of the fish were concentrated on the left side of the glass tank in the following hours (5–6 h, Figure 3f, and 9–10 h, Figure 3g). This situation became clear in Figure 3h, which covers all 10-hour data, with a decrease in the frequency of the fish movements in the bottom areas of the glass tank and a sparser distribution. The data for the glass tank to which 200 mg·lt^−1^ ammonium chloride was added are presented in Figure 3i–l. As a result of increasing the applied dosage, some significant changes occurred in the movements of the fish. While the fish were moving around freely in every part of the glass tank in the first hours (1–2 h, Figure 3i), it was observed that the movements were concentrated on the left side of the glass tank in the following hours (5–6 h, Figure 3j, and 9–10 h, Figure 3k). In the glass tank where 200 mg·lt^−1^ was applied, it was clearly shown that the movement area was narrowed in all-hour intervals and that the fish exhibited stressful behavior with a sparser distribution in other areas (Figure 3l). The data of the glass tank to which 400 mg·lt^−1^ of ammonium chloride was added are presented in Figure 3m–p. As can be seen, when the applied dosage was increased to 400 mg·lt^−1^, it was determined that the fish were very clearly clustered in a certain area in the first hours (1–2-hour intervals) (Figure 3m). As time progressed (5–6 h), the movements of the fish began to concentrate in certain areas. This indicates that the effect of ammonium chloride started, and the fish were moving towards less stressful areas (Figure 3n). In the last-hour intervals (9–10 h), it was determined that the movements of the fish were severely restricted and concentrated in a certain area close to the water surface (Figure 3o). This situation is seen in Figure 3p, which covers all 10-hour data, where the long-term effect of ammonium chloride creates a more pronounced stress on the behavior of the fish compared to other dose amounts and is completely absent in other regions.

**Figure 4:**
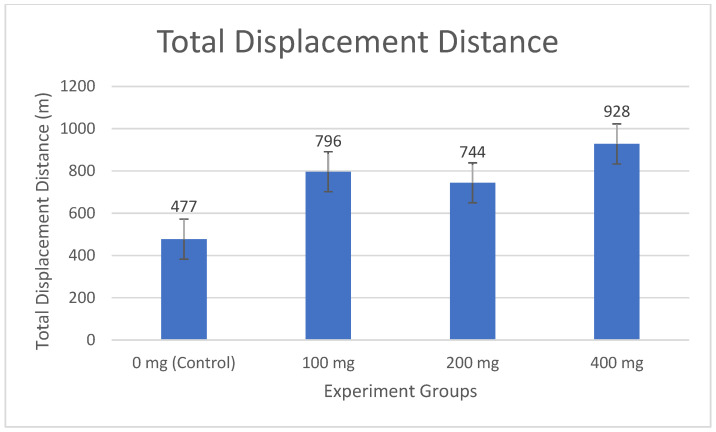
Total displacement distance of fish in experimental groups for 10 h (*n* = 2 fish per group).

Increasing ammonia concentrations restricted fish movement areas (Figure 3), with clustering observed at 100 mg·lt^−1^ (796 m), intensified at 200 mg·lt^−1^ along the tank’s left edge (744 m), and severe restriction near the water surface at 400 mg·lt^−1^ (928 m). Concurrently, total movement distance and irregularity increased compared to the control (0 mg·lt^−1^) (477 m), reflecting more frequent and erratic movements within confined areas (Figure 4, Table 3).

In the study, while the fish in the 0 mg·lt^−1^ group traveled an average of 477 m, this value increased to 796 m in the 100 mg·lt^−1^ group, 744 m in the 200 mg·lt^−1^ group, and 928 m in the 400 mg·lt^−1^ group (Figure 4). The linear increase in total movement distance as the ammonia concentration increased showed that the fish became more active under stress and made more frequent and irregular movements in a limited area. While the least movement was in the 0 mg·lt^−1^ group, it was observed that the total movement amount increased depending on the dosage in the other groups.

**Movement Speed Analysis**

Table 3 shows the mean displacement speeds (mm·s^−1^), standard deviations, and movement ranges of Nile Tilapia fish exposed to different ammonia concentrations (0, 100, 200, and 400 mg·lt^−1^). The control group (0 mg·lt^−1^) had the highest mean displacement speed among all groups, with a mean displacement speed of 21.60 mm·s^−1^, and the movement range ranged between 0 and 108.64 mm·s^−1^. The standard deviation was calculated as ±13.81, indicating that the variability in movement was relatively low and the fish exhibited a regular, homogeneous movement pattern. This indicated that the fish freely used the entire glass tank without ammonia stress and did not show erratic behavior due to stress. The 100 mg·lt^−1^ group had a mean displacement speed of 21.97 mm·s^−1^, which was very close to the control group, and the movement range was determined as 0–89.79 mm·s^−1^. The standard deviation increased to ±15.98, indicating that there was more variety and irregularity in movements compared to the control group. The decrease in maximum speed compared to the control group (from 108.64 to 89.79 mm·s^−1^) suggests that low ammonia concentration may limit the capacity of fish to move at high speeds. This supports the fact that fish movement areas started to cluster in certain areas, namely the left side of the glass tank. The 200 mg·lt^−1^ group had an average displacement speed of 21.17 mm·s^−1^, which was close to the control and 100 mg·lt^−1^ groups, while the range of movement was measured as 0–99.20 mm·s^−1^. The standard deviation was ±15.74, which remained high like the 100 mg·lt^−1^ group, indicating that movements became irregular. The maximum speed was slightly higher (99.20 mm·s^−1^) than the 100 mg·lt^−1^ group (89.79 mm·s^−1^), indicating that the fish occasionally exhibited faster movements under stress at this concentration, but their general movement range was narrowed, concentrated at the left edge of the glass tank. The fact that the speed remained stable in this group may indicate that the fish developed a type of adaptation to moderate ammonia stress. The 400 mg·lt^−1^ group reached the highest level among all groups with an average displacement speed of 25.69 mm·s^−1^ at the highest ammonia concentration, and the movement range showed the widest extent with 0–112.76 mm·s^−1^. The standard deviation was ±15.27, reflecting a similar movement variety with the other ammonia groups, but it was significantly higher compared to the control group. The maximum speed reached 112.76 mm·s^−1^, indicating that fish exhibit sudden and rapid movements under stress, but these movements are generally concentrated in restricted areas such as near the water surface. This confirms that high ammonia concentration seriously affects the behavior of fish and increases their efforts to escape from the toxic environment.

The normality test of the movement data was performed with the Kolmogorov–Smirnov test, and it was determined that the data did not conform to the normal distribution (*p* < 0.05). The differences between the groups were analyzed using the Kruskal–Wallis test, and a significant difference was detected (*p* < 0.05). The results of the post hoc Dunn test (Bonferroni-corrected) are given in Table 4. No statistically significant difference was detected between the control and 100 mg·lt^−1^ groups (*p* > 0.05). Significant differences were detected between the control group and the 200 mg·lt^−1^ and 400 mg·lt^−1^ groups (*p* < 0.05). Differences were also observed between the 100 mg·lt^−1^ group and the 200 mg·lt^−1^ and 400 mg·lt^−1^ groups (*p* < 0.05). A significant difference was detected between the 200 mg lt^−1^ and 400 mg·lt^−1^ groups regarding speed (*p* < 0.05). The 400 mg·lt^−1^ group was distinguished from all other groups by the highest average speed and wide range of motion, which statistically proved the dominant effect of high ammonia concentration on fish behavior. To track fish movements, images taken from the side and top of the glass tank were examined with various methods. The working accuracy of the software used in the study was performed manually with the developments made on the software developed by the researchers. This process provided a fast and reliable method when analyzing the accuracy of the data set. In this study, because of the manual verification processes of fish detection on each selected image, it was determined that the software detected fish with an accuracy rate of 91.40% (36.000 images for each group). This rate shows that the system can track fish movements reliably.

## 4. Discussion

Ref. [20] trained the Faster R-CNN and YOLO-V3 models on fish images to determine the effect of ammonia on goldfish, compared the accuracy of these models in detecting fish, and drew fish trajectories in an environment where ammonia was added. In this study, the effects of ammonia on tilapia were analyzed using an algorithm developed with the OpenCV software library and Python codes in the Visual Studio Code software development environment. It was clearly demonstrated that stress increased, and fish behavior changed over time with color transitions. Ammonium chloride (NH_4_Cl) is a common pollutant in aquatic environments and has been shown to cause various adverse effects in fish, and it can even lead to physical damage, behavioral changes, and death in fish [46,47,48]. Ref. [49] reported that ammonium chloride exposure increases physiological stress in fish and that this situation can be measured with biomarkers. According to [50], low concentrations may not harm or kill fish in short-term exposure, but even at the same concentrations, toxic effects can harm or kill them, depending mainly on the duration of exposure, dissolved ions, pH, water temperature, and size of the fish [47,48,50]. According to [7], increased ammonia load can lead to ionic imbalance, hyper-excitability, behavioral changes, convulsions, and death.

In this study, no deaths were encountered in the fish. But, as other researchers have stated in the studies that ammonia has negative reactions on fish, this study also detected negative effects on fish behavior. Ref. [20] applied 100 mg·lt^−1^, 200 mg·lt^−1^, and 400 mg·lt^−1^ ammonia concentrations to goldfish and reported that the fish died at the ninth hour at the concentration applied at 600 mg·lt^−1^. Similarly, in this study, the displacement speeds (mm·s^−1^), standard deviations, and movement ranges of Nile Tilapia (*O. niloticus*) fish when exposed to different ammonia concentrations (0, 100, 200, and 400 mg·lt^−1^) and the effects of ammonia stress on their mobility levels are revealed. The 600 mg·lt^−1^ dose was not applied because it could be lethal.

The observed restriction in movement area alongside increased movement distance (e.g., 928 m at 400 mg·L^−1^ vs. 477 m in control) suggests that ammonia stress induces erratic, repetitive movements within confined spaces, possibly as an escape response. This aligns with Xu et al. (2020), who reported similar behavioral shifts in goldfish under ammonia stress, and may reflect physiological stress responses, such as elevated cortisol levels [20].

In their study, Ref. [51] stated that zebrafish (*Danio rerio*) wandered homogeneously in the tank without a specific movement pattern in the resting state. In this study, when the data of all control groups without ammonium chloride were examined, it was observed that the fish spread to a wider area where they were in every point of the glass tank. This may mean that they maintained their normal behavior patterns and reflected the natural behavior of the fish in a healthy and stress-free environment. No significant difference was detected between the control groups, and this was also supported by statistical analysis. In the data of the glass tank with 100 mg·lt^−1^ ammonium chloride, it was determined that the fish were in every point of the glass tank in the first hours, while the movements of the fish were clustered on the left side of the glass tank in the later hours. In all hourly data, it was observed as a dilution at the bottom of the glass tank. But, even at an ammonia concentration of 100 mg·lt^−1^, the fact that the movements of the fish were clustered in a certain area (left side) over time indicates that ammonia can cause stress even at low levels. Ref. [20] reported that the movement area narrowed at a similar concentration in goldfish, which shows that tilapia is also sensitive to low ammonia levels. At 200 mg·lt^−1^, there was a significant change in the movements of the fish. While the fish were observed at every point of the glass tank in the first hours, dense clustering was observed on the left edge of the glass tank in the later hours. This shows that even at low concentrations, ammonium chloride can cause stress on fish and limit their movement areas. Ref. [37] reported that long-term exposure to low levels of ammonia slows down the growth rate, reduces the feed conversion rate, and causes histopathological changes to occur in liver and gill tissues and may have long-term negative effects on fish health and growth. In the glass tank where 400 mg·lt^−1^ ammonium chloride was applied, fish showed obvious regional concentrations. As time progresses, the effect of ammonium chloride is more serious, restricting the movements of fish and limiting their movements under stress by staying in a certain area. Contrary to [20], who reported reduced movement in goldfish under ammonia stress, our findings show increased movement distance (e.g., 928 m at 400 mg·lt^−1^ vs. 477 m in control) and speed (Table 3). This discrepancy may stem from species-specific responses, as Nile Tilapia may exhibit heightened escape behavior under stress compared to goldfish. Alternatively, the 10-hour exposure duration in our study might capture acute stress responses, whereas longer exposures could lead to reduced activity, as noted in some studies [36]. At 400 mg·lt^−1^, the increased movement distance (928 m) and speed (25.69 mm·s^−1^) (Table 3) suggest that high ammonia concentrations induce acute stress responses, likely reflecting escape attempts rather than adaptive slowing of movements. This heightened activity may increase energy expenditure and reduce survival chances in toxic environments, which is consistent with physiological stress effects reported by [38].

When the locomotion of fish in ammonia concentrations of 100, 200, and 400 mg·lt^−1^ and the control group (0 mg·lt^−1^) was compared, the mean displacement speed in the control group was 21.60 mm·s^−1^, the standard deviation was ±13.81, and the range of movement was 0–108.64 mm·s^−1^, indicating regular movements. In ammonia groups, the speeds were measured as 21.97 mm·s^−1^, 21.17 mm·s^−1^, and 25.69 mm·s^−1^, respectively, and it was observed that fish were more active, especially at 400 mg·lt^−1^. At 100 and 200 mg·lt^−1^, the standard deviations (±15.98, ±15.74) increased, confirming the irregularity of movement, while at 400 mg·lt^−1^, the wide range of movement (0–112.76 mm·s^−1^) confirmed the high mobility. Ref. [20] reported that high ammonia doses increased the movement speed; the findings at 400 mg·lt^−1^ support this observation.

In this study, when the data of all control groups without ammonium chloride supplementation were examined, they exhibited low movement diversity with an average displacement speed of 21.60 mm·s^−1^, ±13.81 standard deviation, and 0–108.64 mm·s^−1^ movement range. This confirms that the fish used the entire glass tank homogeneously without ammonia stress and showed a regular movement pattern. The low standard deviation indicates that the movements were predictable, and there were no stress-related irregularities. Ref. [52] reported that tilapia exhibited constant movement patterns without stress factors. The control group findings in this study support this observation. Ref. [36] stated that metabolic scope was optimal in tilapia in the absence of stress; low movement diversity can be interpreted as a behavioral reflection of this physiological balance. It may mean that it reflects the natural behavior of the fish in a healthy and non-stressed environment. No significant difference was detected between the control groups, and this was also supported by statistical analysis.

In this study, at a concentration of 100 mg·lt^−1^, the average displacement speed remained close to the control group at 21.97 mm·s^−1^, but the standard deviation increased to ±15.98, and the movements became more irregular. The range of movement narrowed to 0–89.79 mm·s^−1^, and the maximum speed decreased compared to the control group (from 108.64 mm·s^−1^ to 89.79 mm·s^−1^), suggesting that the capacity of the fish to move at high speeds decreased. With this it can be said that low ammonia concentrations slightly affected locomotion but did not create a significant stress response. Ref. [20] reported that low ammonia doses in goldfish minimally changed the movement speed; a similar tolerance was observed in tilapia. But, [51] stated that low-stress factors increased movement diversity in zebrafish; the high standard deviation supports this finding. These results suggest that 100 mg·lt^−1^ may be the upper limit for fish welfare, and this level should be carefully monitored in water quality management.

Kruskal–Wallis and post hoc Dunn tests showed that the displacement speed of *O. niloticus* fish at 400 mg·lt^−1^ ammonia concentration was significantly different from the control (0 mg·lt^−1^), 100 mg·lt^−1^, and 200 mg·lt^−1^ groups (*p* < 0.05). This indicates that ammonia concentrations between 200–400 mg·lt^−1^ significantly increased fish locomotion and triggered behavioral changes. The absence of any difference between the control and 100 mg·lt^−1^ (*p* > 0.05) groups supports that tilapia developed tolerance to low concentrations in the short term [36]. The increase in movement irregularity and space restriction at concentrations of 200 mg·lt^−1^ and above reflects the effect of ammonia toxicity. The high locomotion at 400 mg·lt^−1^ is associated with this physiological stress. These results are consistent with those of [38] which reported that the interaction of ammonia and salinity impairs antioxidant defense and weakens immunity. This image processing-based method provides a significant advantage over traditional blood sampling methods by offering the opportunity to monitor fish behavior in real time and non-invasively [16]. The algorithm, which operates with an accuracy rate of 91.40%, provides a reliable basis for the development of automation in aquaculture. Ref. [31] stated that real-time video analysis optimizes feeding processes; similarly, this study provides an applicable tool for the early detection of ammonia stress and the improvement of water quality management. Ref. [15] stated that *C. carpio* fish immediately respond to increasing ammonia levels by diving to the bottom of the tank and staying there for a while to adapt. Ref. [36] reported that short-term acute ammonia exposure to high ammonia levels in Nile Tilapia may have significant effects on fish physiology, such as increased heart rate, decreased metabolic scope, and increased stress hormones. Ref. [38] emphasized that ammonia exposure and salinity changes together cause more severe effects than ammonia exposure alone, disrupt antioxidant defense systems, weaken the immune system, and may decrease growth performance, as well as the complex effects of the interaction of environmental factors on fish health. Ref. [20] stated that the effect of different ammonia concentrations on fish behavior and the three-dimensional movement route of fish can be drawn thanks to the deep learning approach, and the behavior of fish can be analyzed in more detail. Fish behavior may reflect changes in the culture environment or the impact of diseases. Ref. [20] emphasized that this method is simple, effective, and non-invasive; it not only provides an innovative approach to investigating fish behavior but also provides an effective and applicable method for monitoring the marine culture environment. These observations indicate that the presence of substances such as ammonium chloride in water can seriously affect the behavior and health of fish, which is a critical effect of water quality on fish health and a crucial factor to be considered for preventing chemical contamination in glass tank environments. This indicates that the effect of ammonium chloride on fish increases over time, and water quality should be continuously monitored and corrected, if necessary, as it is critical for water quality management to understand the effects of water quality changes on fish health and behavior.

## 5. Conclusions

This study demonstrated that increasing ammonium chloride concentrations (0, 100, 200, 400 mg·lt^−1^) significantly altered individual Nile Tilapia (*Oreochromis niloticus*) behavior. Non-invasive image processing, using the Optical Flow Farneback algorithm (91.40% accuracy), revealed that control fish moved uniformly (477 m), while 100 mg·lt^−1^ increased clustering (796 m), 200 mg·lt^−1^ confined edge movements (744 m), and 400 mg·lt^−1^ caused severe surface restriction with erratic activity (928 m). Statistical analyses confirmed distinct changes at 400 mg·lt^−1^, with no difference between the control and 100 mg·lt^−1^, indicating tolerance to low concentrations. Concentrations above 100 mg·lt^−1^ impaired fish welfare, emphasizing ammonia management in aquaculture. Future research should explore group behaviors, chronic exposures at <1 mg·lt^−1^ NH_4_, and anomaly detection methods to enhance automation. This approach validates non-invasive monitoring for improving fish health and aquaculture efficiency.

## Figures and Tables

**Table 1:** Length and weight of the fish used in the experiments.

	Experiment 1 (100 mg·lt^−1^)		Experiment 2 (200 mg·lt^−1^)		Experiment 3 (400 mg·lt^−1^)	
	Glass Tank 1	Glass Tank 2	Glass Tank 1	Glass Tank 2	Glass Tank 1	Glass Tank 2
**Length (cm)**	13	14	14.2	13.5	15.2	15.1
**Weight (gr)**	33.4	36.8	41.2	37	49.1	49.5

**Table 2:** Water parameters measured during the experiments.

Water Parameters				pH	Temperature (°C)	O_2_ (mg·lt^−1^)	NO_2_ (mg·lt^−1^)	NO_3_ (mg·lt^−1^)	NH_3_ (mg·lt^−1^)	NH_4_ (mg·lt^−1^)
**Experiment 1 (100 mg·lt^−1^)**	**Glass Tank 1**	**Day 1**	**Morning**	7.55	24.21	7.07	0	0	0.5	0.5
			**Evening**	7.91	24.33	7.05	0	0	0.5	0.5
		**Day 2**	**Morning**	7.74	24.58	7.71	0	0	>10	>10
			**Evening**	8.25	24.23	7.65	0	0	>10	>10
	**Glass Tank 2**	**Day 1**	**Morning**	7.52	24.17	7.17	0	0	0.5	0.5
			**Evening**	7.98	24.26	7.06	0	0	0.5	0.5
		**Day 2**	**Morning**	7.78	24.42	7.72	0	0	>10	>10
			**Evening**	8.24	24.17	7.59	0	0	>10	>10
**Experiment 2 (200 mg·lt^−1^)**	**Glass Tank 1**	**Day 1**	**Morning**	7.59	24.29	7.15	0	0	0.5	0.5
			**Evening**	7.92	24.54	7.08	0	0	0.5	0.5
		**Day 2**	**Morning**	7.77	24.43	7.17	0	0	>10	>10
			**Evening**	8.26	24.38	7.04	0	0	>10	>10
	**Glass Tank 2**	**Day 1**	**Morning**	7.53	24.32	7.18	0	0	0.5	0.5
			**Evening**	7.95	24.49	7.06	0	0	0.5	0.5
		**Day 2**	**Morning**	7.72	24.27	7.16	0	0	>10	>10
			**Evening**	8.29	24.15	7.04	0	0	>10	>10
**Experiment 3 (400 mg·lt^−1^)**	**Glass Tank 1**	**Day 1**	**Morning**	7.57	24.46	7.41	0	0	0.5	0.5
			**Evening**	7.93	24,39	7.34	0	0	0.5	0.5
		**Day 2**	**Morning**	7.79	24.32	7.15	0	0	>10	>10
			**Evening**	8.21	24.51	7.11	0	0	>10	>10
	**Glass Tank 2**	**Day 1**	**Morning**	7.51	24.28	7.23	0	0	0.5	0.5
			**Evening**	7.97	24,36	7.17	0	0	0.5	0.5
		**Day 2**	**Morning**	7.75	24.19	7.18	0	0	>10	>10
			**Evening**	8.22	24.45	7.09	0	0	>10	>10

**Table 3:** Movement levels of fish in groups (average speed, standard deviation, min–max range) displacement distances.

Displacement (mm·s^−1^)				
Groups	Minimum	Maximum	Mean	Standard Deviation
**0 mg·lt^−1^**	0	108.64	21.60	±13.81
**100 mg·lt^−1^**	0	89.79	21.97	±15.98
**200 mg·lt^−1^**	0	99.20	21.17	±15.74
**400 mg·lt^−1^**	0	112.76	25.69	±15.27

**Table 4:** Post hoc Dunn test results (speed differences between groups).

	0 mg·lt^−1^	100 mg·lt^−1^	200 mg·lt^−1^	400 mg·lt^−1^
**0 mg·lt^−1^**	X *p* = 1.000000	No difference *p* = 1.000000	Difference 2.906691 × 10^−2^	Difference 3.176312 × 10^−23^
**100 mg·lt^−1^**	No difference *p* = 1.000000	X *p* = 1.000000	Difference 7.724616 × 10^−03^	Difference 1.763345 × 10^−21^
**200 mg·lt^−1^**	Difference 2.906691 × 10^−2^	Difference 7.724616 × 10^−3^	X *p* = 1.000000	Difference 2.046009 × 10^−37^
**400 mg·lt^−1^**	Difference 3.176312 × 10^−23^	Difference 1.763345 × 10^−21^	Difference 2.046009 × 10^−37^	X *p* = 1.000000

## Data Availability

Data are available upon request.
